# Psychological and Psychopharmacological Interventions in Psychocardiology

**DOI:** 10.3389/fpsyt.2022.831359

**Published:** 2022-03-16

**Authors:** Kai G. Kahl, Britta Stapel, Christoph U. Correll

**Affiliations:** ^1^Department of Psychiatry, Social Psychiatry and Psychotherapy, Hannover Medical School, Hanover, Germany; ^2^Department of Psychiatry Research, The Zucker Hillside Hospital, Glen Oaks, NY, United States; ^3^Department of Psychiatry and Molecular Medicine, Donald and Barbara Zucker School of Medicine at Hofstra/Northwell, Hempstead, NY, United States; ^4^Department of Child and Adolescent Psychiatry, Charité – Universitätsmedizin Berlin, Berlin, Germany

**Keywords:** psychocardiology, psychological intervention, psychopharmacological intervention, cardiovascular disease, antidepressant, antipsychotic, psychotherapy

## Abstract

Patients with mental disorders have an increased risk to develop cardiovascular disease (CVD), and CVD are frequently comorbid with especially adjustment, anxiety and depressive disorders. Therefore, clinicians need to be aware of effective and safe psychological and pharmacological treatment strategies for patients with comorbid CVD and mental disorders. Cognitive behavioral therapy and third-wave of cognitive-behavioral therapy are effective for patients with CVD and mental disorders. Internet-based psychological treatments may also be considered. In more severe cases, psychopharmacological drugs are frequently used. Although generally well tolerated and efficacious, drug- and dose-dependent side effects require consideration. Among antidepressants, selective serotonin reuptake inhibitors, selective serotonin and noradrenalin reuptake inhibitors, and newer antidepressants, such as mirtazapine, bupropion, agomelatine, and vortioxetine, can be considered, while tricyclic antidepressants should be avoided due to their cardiac side effects. Mood stabilizers have been associated with arrhythmias, and some first- and second-generation antipsychotics can increase QTc and metabolic side effects, although substantial differences exist between drugs. Benzodiazepines are generally safe in patients with CVD when administered short-term, and may mitigate symptoms of acute coronary syndrome. Laboratory and ECG monitoring is always recommended in psychopharmacological drug-treated patients with CVD. Presence of a heart disease should not exclude patients from necessary interventions, but may require careful risk-benefit evaluations. Effectively and safely addressing mental disorders in patients with CVD helps to improve both conditions. Since CVD increase the risk for mental disorders and vice versa, care providers need to screen for these common comorbidities to comprehensively address the patients’ needs.

## Introduction

Severe mental disorders, such as major depressive disorder (MDD), pose a higher risk for the development of cardiovascular disease (CVD), and vice versa (1, 2). In particular, adjustment disorder, anxiety disorders and MDD are more frequent in CVD patients compared to the general population (3, 4). The comorbidity of CVD and mental disorders is associated with worse treatment outcomes and decreased quality of life (3, 5–10). The high prevalence of comorbid severe mental diseases and CVD has been attributed to several contributing factors. In this regard, dysregulation of biological systems as well as adverse health behaviors and insufficient economic and social support have been implied (11–15). Overall, severe mental diseases were found to be associated with pathological alterations in stress response systems, with systemic low-grade inflammation, with abnormalities in heart rate variability, and with reduced baroreflex sensitivity (16–18), and of note similar processes were found to profoundly impact metabolic pathways and subsequent development and progression of CVD (19, 20). Additionally, tobacco and alcohol consumption, a sedentary lifestyle, and poor diet, factors known for their contribution to the onset and progression of CVD, are common in patients with severe mental disorders (11, 12). Finally, patients suffering from mental disorders appear less likely to adhere to treatment regimens and were frequently found to have insufficient access to physical health care (21, 22).

Given the significant impact of mental disorders on the outcome of CVD patients, treatment interventions for comorbid patients are clinically important.

According to national and international guidelines, (23, 24) mental disorders within and without the context of an underlying physical illness can be treated using psychological and psychopharmacological interventions. However, iatrogenic effects of psychotropic medications have been described (1, 25). In this regard, the safety and efficacy of psychotropic medications in patients with CVD varies greatly, depending on drug- as well as disease type (26, 27). Consequently, precise knowledge regarding potential adverse effects of frequently used psychotropic medications, i.e., antipsychotics, antidepressants, and mood stabilizers, on parameters of physical health are indispensable in the treatment of comorbid mental disorders and CVD.

## Methods

Narrative review of practical clinical considerations when treating patients with cardiovascular and mental disorders pharmacologically and psychologically for their mental disorders.

## Results

### Psychopharmacological Interventions

#### Selective Serotonin Reuptake Inhibitors

National and international guidelines recommend the use of selective serotonin reuptake inhibitors (SSRIs) for the treatment of MDD and anxiety disorders (23, 24, 28–30). SSRIs are well tolerated in general, and beneficial effects of SSRI treatment in patients with depression following acute coronary syndrome regarding re-hospitalization rates have been shown in a dedicated meta-analysis (31). However, SSRIs pose the risk of cardiovascular side effects in a dose- and substance dependent manner. In principle, all SSRIs can lead to some QTc prolongation. However, especially citalopram (and to a much lesser degree, its enantiomer es-citalopram) pose a relatively higher risk compared to other SSRIs (i.e., fluoxetine, fluvoxamine, paroxetine, sertraline). A meta-analysis revealed that SSRIs as a class increase QTc interval for an average of 6 ms (32). Although on average, this is not clinically relevant, subgroups of patients may experience clinically relevant QTc prolongation. Along with QTc prolongation, the risk for the development of ventricular arrhythmia and Torsades-de-Pointes (TdP) is increased (33–37). Most cases of SSRI-induced QTc interval prolongation and subsequent arrhythmia were observed in older patients above 65 years of age. Further risk factors comprise low potassium levels, female sex, polypharmacy, pre-existing cardiovascular disorders, pre-existing liver and/or kidney disease, and high doses of SSRIs (33, 38, 39). As mentioned above, among the SSRIs, this risk is highest for citalopram and es-citalopram. The United States Food and Drug Administration (FDA) as well as the European Medicines Agency (EMA) accordingly gave a safety warning, recommending doses not higher than 40 mg/day for citalopram and 20 mg/day for es-citalopram (40, 41). The relative risk of QTc prolongation with frequently used antidepressant drugs is summarized in Table 1.

**TABLE 1:** Relative risk of QTc prolongation with frequently used antidepressant drugs.

Substance	Risk of QTc prolongation
citalopram, es-citalopram, TCAs (particularly amitriptyline)	+
fluoxetine, fluvoxamine, mirtazapine, sertraline, venlafaxine	(+)
agomelatine, bupropion, duloxetine, milnacipran, paroxetine, reboxetine, vortioxetine	(±)

*+, existent; (+), low; (±), very low.*

Additionally, among different classes of antidepressants, SSRI treatment was found to be associated with the highest risk for hyponatraemia, brought forward by a drug-induced syndrome of inappropriate antidiuretic hormone (ADH) secretion (SIADH) (42). While conclusive evidence for differences of individual SSRIs regarding SIADH risk are lacking (43), two larger studies reported lower risks for paroxetine and sertraline compared to other SSRIs including fluoxetine, citalopram, and es-citalopram (44–47). Moreover, elevated SIADH risk under SSRI treatment has been found in the geriatric population and especially in concomitant (thiazide) diuretics (42, 48).

Further side effects of SSRIs with possible cardiovascular relevance are orthostatic hypotension, primarily in elderly patients (49–52), and impaired platelet activation and aggregation, possibly leading to an increased risk for intracerebral hemorrhages (45, 53–56). Therefore, SSRI treatment should be given carefully in patients with underlying hemostatic abnormalities and on anticoagulation treatment; additionally, non-steroidal anti-inflammatory drugs (NSAID) should not be given in SSRI treated patients (32, 57–59).

#### Serotonin Norepinephrine Reuptake Inhibitors

Serotonin norepinephrine reuptake inhibitors (SNRIs), particularly venlafaxine, can increase heart rate and blood pressure due to their noradrenergic effects (60–65). Among the SNRIs, venlafaxine and duloxetine may be associated with a possible risk of QTc prolongation, particularly in overdose situations (66, 67). Milnacipran, levomilnacipran and reboxetine have sometimes been associated with hypotension, particularly when administered at higher dosages (68–70). Albeit less frequent compared to SSRIs, SNRI treatment can lead to electrolyte disturbances, particularly hyponatremia (25, 42, 44, 71).

#### Tricyclic Antidepressants

Strong cardiovascular side effects are described with tricyclic antidepressants (TCAs) due to their anticholinergic and chinidine-like properties. In particular, cardiac conduction abnormalities, such as atrioventricular block, QTc prolongation, atrial fibrillation, and ventricular tachycardia, have been described (72–75). Further, the use of TCAs has been associated with acute myocardial infarction and a higher risk of stroke compared to SSRIs (75).

#### Other Frequently Used Antidepressants

##### Agomelatine

Agomelatine is a melatonin agonist with antidepressant properties. To date, no evidence of cardiovascular risk has been reported (76). Of note, liver enzymes should be controlled frequently due to its dose-dependent liver toxic properties.

##### Mirtazapine

Mirtazapine acts as specific serotonergic and noradrenergic antidepressant drug, with a relatively favorable cardiovascular side effect profile (73). However, significant weight gain and sedation have been frequently, and orthostatic hypotension has also sometimes been described (25, 77). A prospective multicenter study including 2,177 patients with myocardial infarction and depression found no significant effect of mirtazapine on cardiac event rate at 18 months (78).

##### Vortioxetine

Vortioxetine, a multimodal antidepressant and serotonin transporter inhibitor, seems to have a favorable cardiovascular side effect profile (79, 80). Besides antidepressant efficacy, pro-cognitive and anti-inflammatory properties have been described (81).

##### Bupropion

Bupropion at therapeutic dosages has not been associated with adverse effects on heart rate, blood pressure, and other cardiovascular parameters (82). In patients with CVD, bupropion was found to have a favorable effect on depressive symptoms (83), and was also observed to be safe in the post-myocardial infarction period when given at therapeutic dosages (84). Although hypertension and tachycardia have been described (85), bupropion does not appear to induce severe cardiovascular events, even in overdose.

##### Recommendations

A substantial body of evidence indicates that the cardiovascular safety profile of newer-generation antidepressants is significantly improved compared to TCAs (76). Nevertheless, regular monitoring concerning blood pressure, heart rate, ECG, body weight, and routine laboratory parameters (such as potassium levels) is generally recommended.

Selective serotonin reuptake inhibitors and SNRIs are generally considered safe in the treatment of patients with comorbid CVD when given within therapeutic limits. Due to the potential to increase the QTc interval (SSRIs and SNRIs) and heart rate/blood pressure (SNRIs), regular ECG and blood pressure monitoring is recommended. A combination of SSRIs with drugs that prolong QTc interval or that are associated with an increased bleeding risk (i.e., NSAIDs) should be avoided.

Newer antidepressant drugs (agomelatine, vortioxetine, mirtazapine, and bupropion) are generally considered safe when given within therapeutic dose limits. Albeit literature appears limited, the risk of QTc prolongation was found to be low for the majority of newer antidepressants (66). Weight monitoring (especially with mirtazapine) and blood pressure monitoring (especially with venlafaxine, duloxetine and bupropion) are recommended. For agomelatine, liver enzyme monitoring is recommended due to its propensity to significantly increase liver enzymes in up to 0.8% of treated patients.

Although substance- and dose-dependent, TCAs should be avoided in patients comorbid with CVD and mental disorders due to their potentially strong cardiovascular side effects.

#### First-Generation (Typical) and Second-Generation (Atypical) Antipsychotic Drugs

First- and second-generation antipsychotic drugs are used to treat patients with psychotic disorders, particularly schizophrenia (86, 87), and patients with bipolar disorders (88–90). Furthermore, several second-generation antipsychotics are recommended as augmentation strategy in treatment-resistant depression (91).

The increased cardiovascular morbidity and mortality observed in patients that receive antipsychotic drugs has been attributed to several factors, including an increased rate of unhealthy lifestyle (smoking, physical inactivity, and unhealthy eating habits), suboptimal screening and secondary preventive medical measures, and direct and indirect effects of antipsychotic medications (1, 92–94).

First-generation and, especially, second-generation antipsychotics have been associated with QTc prolongation and/or TdP (93, 95–109), as well as sudden cardiac death due to cardiac arrhythmia (95–97, 110, 111). More specifically, QTc prolongation has been reported for some first-generation antipsychotics (i.e., pimozide, IV haloperidol) and some low-potency phenothiazines (especially thioridazine, melperone), which were found to be associated with the greatest risk (112–114). Similarly, some second-generation antipsychotics (i.e., sertindole, iloperidone, amisulpride, ziprasidone) have been associated with OTc interval prolongation. Although a direct risk comparison is complicated by differences in study methodologies (96), especially ziprasidone appears to carry a higher risk for clinically meaningful increases in QTc interval (86, 114, 115). A summary regarding the relative risk of QTc prolongation with frequently used antipsychotic drugs is provided in Table 2. As with QTc prolongation with antidepressant drugs, further risk factors have to be considered, such as female sex, hypopotassemia, hypomagnesemia, higher age, and pre-existent cardiac diseases (116). Polypharmacy and the combination of drugs with the potential to alter cardiac conduction also enhance the risk of QTc prolongation compared to antipsychotic monotherapy (105). Some first- and second-generation antipsychotics are associated with an increased risk for cortical venous thrombosis or pulmonary embolism (117–131). Overall, the risk for hospitalization appears higher for second-generation antipsychotics: quetiapine (hazard ratio 2.68) > risperidone (1.98) > olanzapine (1.87) = clozapine (1.87) > phenothiazines (1.03) or other first-generation agents (0.98) (117).

**TABLE 2:** Relative risk of QTc prolongation with frequently used antipsychotic drugs [modified according to Refs. (132, 86)].

Substance	Risk of QTc prolongation
Thioridazine, pimozide, sertindole, haloperidol (i.m., IV, high dose), iloperidone, melperone, amisulpride, ziprasidone	+++
Chlorpromazine, levomepromazine, quetiapine, sulpiride	++
Haloperidol (p.o.), fluphenazine, perphenazine, promethazine, pipamperone, chlorprothixene, olanzapine, risperidone, asenapine, clozapine	+
Aripiprazole, brexpiprazole, cariprazine, lurasidone, paliperidone, lumateperone	±

*+++, high (>20 ms); ++, moderate (10–20 ms); +, low (<10 ms); ±, very low (< 10 ms, potentially not higher than placebo).*

##### Metabolic Syndrome and Type-2 Diabetes Mellitus

Increased blood lipid and glucose levels have been reported with several second-generation and some low-potency first-generation antipsychotics (93, 94, 133–135). Among these, clozapine and olanzapine as well as chlorpromazine and thioridazine have been associated with an increased risk for metabolic disturbances such as glucose disturbance, lipid changes, and in some cases with acute ketoacidosis (93, 94, 133, 136). Further, second-generation antipsychotics have been reported to increase body weight, with clozapine and olanzapine bearing the greatest risk (93). According to a systematic review of randomized, placebo-controlled studies, the risk of weight gain was reported as follows: clozapine and olanzapine > risperidone > quetiapine > aripiprazole > ziprasidone (93).

Increased blood pressure and orthostatic hypotension have been reported in the context of antipsychotic treatment. Concerning blood pressure, around one third of patients with a psychotic disorder presents with elevated blood pressure; however, it is unclear whether this effect is due to antipsychotic medication or to formerly undetected metabolic syndrome and its components (136).

Orthostatic hypotension, most frequently observed in response to treatment with first-generation antipsychotics, such as phenothiazine, chlorpromazine, and thioridazine, is mostly attributed to their antagonistic action on adrenergic receptors (137).

##### Recommendations

The use of antipsychotic medications is in general safe in patients with comorbid CVD and mental disorder, although caution is necessary concerning direct (ECG alterations) and indirect (cardiometabolic and thromboembolic) side effects of certain drugs. For most psychiatric diseases, second-generation antipsychotics are currently recommended, rather than first-generation antipsychotics. Within the group of second-generation antipsychotics, the risk for cardiometabolic side effects (i.e., weight gain, hyperlipidemia, insulin resistance) differs substantially between drugs and is highest with the use of clozapine and olanzapine. In patients that present with, or display an increased risk for metabolic syndrome, drugs with lower metabolic side effects (i.e., aripiprazole, brexpiprazole, cariprazine, lurasidone, or lumateperone) should be considered.

Patients should be monitored closely for metabolic side effects, particularly in the first year of treatment. ECG monitoring should take place before and during antipsychotic treatment in patients with clinical risk factors for cardiac arrhythmias, i.e., family history of early cardiac death (<55 years old), personal history of structural heart defects, dizziness and, especially, syncope upon exertion, palpitations at rest (138). In CVD patients treated with clozapine, particular attention should be paid to anticholinergic side effects and the risk for myocarditis (139).

According to recommendations detailed in a consensus document by the American Diabetes Association (ADA) and American Psychiatric Association (APA), weight should be monitored monthly for the first three months and then quarterly, fasting blood glucose levels should be assessed at baseline, after 3 months, and then annually. A fasting lipid profile should be obtained at baseline, after 3 months, and then every 5th year (140), although the latter recommendation has been amended since, in that lipid levels ought to be monitored at the same time intervals as fasting glucose levels and that HbA1C should also be monitored, as increases occur several years before fasting glucose is elevated, signaling prediabetes or diabetes earlier (94).

#### Benzodiazepines

Benzodiazepines (BZDs) are frequently being prescribed for anxiety disorders, insomnia, and alcohol withdrawal and agitation (141–143). Due to their potential for abuse and their addictive properties, short-term use is recommended (144). Of note, some patients may have paradoxical effects of BZD medication, leading to confusion and an increase in agitation and delirium (145). In the context of CVD, BZDs may play a particular role in calming patients quickly who suffer from chest pain and acute myocardial infarction, reducing cardiac stress (146, 147). The addition of BZDs to standard cardiac medication in patients post myocardial infarction was associated with lower rates of re-infarction (148).

##### Recommendations

Benzodiazepines are generally considered safe in patients comorbid with CVD and mental disorders, when given as short-term acute treatment to relieve anxiety and agitation. Long-term use should be avoided due to inherent risks for iatrogenic BZD addiction, and caution should be given to elderly patients who are at greater risk of falls.

#### Mood Stabilizers

Mood stabilizers are given in the context of bipolar disorders to prevent further depressive/manic episodes (88–90), and in MDD as augmentation (91, 149) and, possibly prophylactic treatment (particularly lithium) (150). Lithium has been associated with sinus bradycardia, sinus node dysfunction, atrioventricular lock and ventricular irritability, and sick sinus syndrome is considered a cardiac contraindication for its use (151, 152). Valproic acid is a frequently given drug in epilepsy, and in bipolar disorders. Valproic acid is associated with thrombocytopenia, abnormal platelet function, bleeding risk, and with severe fetal abnormalities, restricting its use in female patients (153–157).

Lamotrigine is an anticonvulsant used in the treatment of bipolar disorder. Lamotrigine is known to bear the risk for toxic epidermal necrolysis (158), which is titration-speed dependent, but is considered safe concerning cardiac complications.

##### Recommendations

Mood stabilizers are generally considered safe concerning cardiovascular side effects in the absence of cardiac conduction delay. Lithium should be avoided in cases of atrioventricular block and sick sinus syndrome.

### Psychological Interventions

Psychotherapy, in particular cognitive behavioral therapy (CBT), is established in the treatment of most major mental disorders, and is also applied to treat patients with mental disorders in the context of an underlying physical disease as well as in cardiometabolic disorders themselves (90, 159–163). In particular, adjustment disorder, anxiety and depressive disorders are frequent comorbidities in CVD, leading to decreased quality of life and in part worsen the disease course of the underlying physical illness (164–166).

Adjustment disorder represents an abnormal stress response to a stressor, such as acute myocardial infarction or chronic heart failure, and is reported to be common in primary care with rates ranging from 1% to 18% (167, 168). MDD also has been associated with a significant bidirectional comorbidity with CVD (169). Compared to MDD, adjustment disorder is almost three times more common in physically ill patients (13% versus 5%) (170) and is also frequently present in conjunction with chronic and potentially life-threatening diseases such as cancer (171). In the context of CVD, rates as high 38% have been reported, particular in acute and potentially life-threatening diseases such as peripartum cardiomyopathy and pulmonary artery hypertension (165, 166).

Different psychological treatments, such as CBT, low intensity psychological interventions, mindfulness-based techniques, metacognitive therapy (172) and e-mental health interventions, have been proposed for the treatment of adjustment disorder. Common components of these treatment strategies are the enabling of patients to reduce or remove the stressor, interventions to improve stress coping, and stress reduction strategies (173). Although there is limited empirical evidence, CBT and CBT-derived strategies are promising treatment interventions in patients with CVD and adjustment disorders (172, 173) and for CVD disorders *per se* (160, 161).

Major depressive disorder and anxiety disorders (particularly panic disorders) are more frequent in patients with CVD compared to the general population (3, 4, 165, 166, 169). Different psychotherapeutic methods are available to treat these disorders including CBT, “third wave” CBT [such as Acceptance and Commitment Treatment (174)] or psychodynamic psychotherapy. CBT is based on the principles established by Aaron T. Beck (175), focusing on dysfunctional believes, maladaptive schemes, and avoidance behavior to counteract mental disorders. Recent meta-analyses revealed that CBT presents an effective treatment to reduce depression and anxiety in patients with CVD (176) and acute coronary syndrome (177).

CBT as a treatment option is often limited by its accessibility. For example, in Germany the time period until a first visit with a psychotherapist takes place lasts up to 3 months, and the duration to the start of psychotherapy may be as long as 6–9 months (178). Therefore, internet-based self-help programs and internet-based psychotherapy have been developed during the last two decades, and efficacy has been proven in the context of anxiety and depressive disorders. These programs are most often based on CBT strategies, and are frequently offered as blended treatments, meaning that a real-life psychotherapist supports the program on demand. A recent meta-analysis demonstrated that internet-based self-help/psychotherapeutic programs have a similar efficacy as face-to-face psychotherapy, although their use has been limited to mild to moderate disease severity (179). Less research has been done for the treatment of patients comorbid with anxiety/depression and CVD. Recently, an internet-based CBT treatment adapted to persons with heart failure was compared to a web-based discussion forum, demonstrating small effects of internet-based CBT (180). In a secondary analysis of the data, improvement of depressive symptoms was demonstrated to be associated with improved autonomous-based self-care (181).

#### Recommendations

Face-to-face CBT and “third wave CBT” interventions have been proven effective in frequent mental disorders that are associated with cardiovascular diseases, and should be considered as treatment options. Due to the mere quantity of CVD patients in need for psychotherapy, and the limited number of psychotherapists, internet-based psychotherapeutic interventions may become a field of growing interest.

## Clinical Summary

Due to the bidirectional association between cardiac and mental diseases, knowledge about treatment interventions and their limitations is important. According to recent research, adjustment disorder, anxiety disorder and MDD are the most frequent mental disorders in patients with CVD; all of them are addressable by psychotherapeutic interventions. Therefore, particularly CBT and its related “third wave CBT” are recommended in patients with CVD and mental disorders of mild to moderate severity. Furthermore, CBT can be recommended in patients with CVD and severe mental disorder as an augmentation of psychopharmacological treatment. If CBT is not accessible, internet-based psychological interventions may also be considered, although more research is needed to prove the effectiveness of internet-based psychotherapy in this population.

In more severe cases, psychopharmacological drugs can be recommended. Although well tolerated and efficacious in general, drug- and dose-dependent side effects have to be considered. Among antidepressants, SSRIs, SNRIs, and newer antidepressants, such as mirtazapine, bupropion, agomelatine and vortioxetine, can be recommended, while TCAs should be avoided due to their cardiac side effects. Mood stabilizers have been associated with arrhythmias, and some first- and second-generation antipsychotic drugs can increase the risk of QTc prolongation and metabolic side effects, although substantial differences exist between individual drugs. BDZs are generally considered safe in CVD when administered short-term. Of note, laboratory and ECG monitoring is always recommended in patients with CDV who are treated with psychopharmacological drugs, starting before the beginning of a drug treatment, and continuing on a regular basis.

Knowledge about effective psychological and safer pharmacological treatment options for mental disorders that are frequently comorbid with CVD can help clinicians optimize the biopsychosocial outcome of patients suffering from both mental and cardiovascular disorders. Importantly, since CVD increases the risk for mental disorders and vice versa, both physical and mental health care providers need to screen for the presence of the common comorbidity to consider the patient in their somato-psychiatric totality, which can help improve overall outcomes.

## Author Contributions

KK: conceptualization, investigation, writing—original draft, and supervision. BS: writing—review and editing. CC: investigation and writing—original draft. All authors contributed to the article and approved the submitted version.

## Conflict of Interest

KK received speaker honoraria by Janssen, Otsuka, Neuraxpharm, EliLilly, Schwabe, Servier, and Trommsdorff/Ferrer; he received an unrestricted grant by Ferrer, ADDISCA. CC has been a consultant and/or advisor to or has received honoraria from: AbbVie, Acadia, Alkermes, Allergan, Angelini, Aristo, Axsome, Cardio Diagnostics, Compass, Damitsa, Gedeon Richter, Hikma, Holmusk, IntraCellular Therapies, Janssen/J&J, Karuna, LB Pharma, Lundbeck, MedAvante-ProPhase, MedInCell, Medscape, Merck, Mindpax, Mitsubishi Tanabe Pharma, Mylan, Neurocrine, Noven, Otsuka, Pfizer, Pharmabrain, Recordati, Relmada, Reviva, Rovi, Seqirus, Servier, SK Life Science, Sumitomo Dainippon, Sunovion, Supernus, Takeda, Teva, and Viatris. He provided expert testimony for Janssen and Otsuka. He served on a Data Safety Monitoring Board for Lundbeck, Relmada, Reviva, Rovi, and Teva. He has received grant support from Janssen and Takeda. He received royalties from UpToDate and is also a stock option holder of Cardio Diagnostics, Mindpax, and LB Pharma. The remaining author declares that the research was conducted in the absence of any commercial or financial relationships that could be construed as a potential conflict of interest.

## Publisher’s Note

All claims expressed in this article are solely those of the authors and do not necessarily represent those of their affiliated organizations, or those of the publisher, the editors and the reviewers. Any product that may be evaluated in this article, or claim that may be made by its manufacturer, is not guaranteed or endorsed by the publisher.
